# The heterogeneity of Parkinson’s disease

**DOI:** 10.1007/s00702-023-02635-4

**Published:** 2023-05-11

**Authors:** Ullrich Wüllner, Per Borghammer, Chi-un Choe, Ilona Csoti, Björn Falkenburger, Thomas Gasser, Paul Lingor, Peter Riederer

**Affiliations:** 1 Department of Neurology, University Clinic Bonn and German Center for Neurodegenerative Diseases (DZNE), 53127 Bonn, Germany; 2 Department of Nuclear Medicine and PET, Aarhus University Hospital, Aarhus, Denmark; 3 Department of Neurology, Klinikum Itzehoe, Robert-Koch-Straße 2, 25524 Itzehoe, Germany; 4 Fachklinik Für Parkinson, Gertrudis Klinik Biskirchen, Karl-Ferdinand-Broll-Straße 2-4, 35638 Leun-Biskirchen, Germany; 5 Department of Neurology, University Hospital Dresden, Fetscherstrasse 74, 01307 Dresden, Germany; 6 Department of Neurology, Hertie-Institute for Clinical Brain Research, University of Tübingen and German Center for Neurodegenerative Diseases (DZNE), Tübingen, Germany; 7 Department of Neurology, School of Medicine, Klinikum Rechts Der Isar, Technical University of Munich, Munich, Germany; 8 Department of Neurology and German Center for Neurodegenerative Diseases (DZNE), Munich, Germany; 9 University Hospital Wuerzburg, Clinic and Policlinic for Psychiatry, Psychosomatics and Psychotherapy, Margarete-Höppel-Platz 1, 97080 Würzburg, Germany; 10 Department of Psychiatry, University of Southern Denmark Odense, J.B. Winslows Vey 18, 5000 Odense, Denmark

**Keywords:** Parkinson’s disease, Phenotypes, Pathophysiology, Personalized medicine, Disease mechanism, Inflammation

## Abstract

The heterogeneity of Parkinson’s disease (PD), i.e. the various clinical phenotypes, pathological findings, genetic predispositions and probably also the various implicated pathophysiological pathways pose a major challenge for future research projects and therapeutic trail design. We outline several pathophysiological concepts, pathways and mechanisms, including the presumed roles of *α*-synuclein misfolding and aggregation, Lewy bodies, oxidative stress, iron and melanin, deficient autophagy processes, insulin and incretin signaling, T-cell autoimmunity, the gut–brain axis and the evidence that microbial (viral) agents may induce molecular hallmarks of neurodegeneration. The hypothesis is discussed, whether PD might indeed be triggered by exogenous (infectious) agents in susceptible individuals upon entry via the olfactory bulb (brain first) or the gut (body-first), which would support the idea that disease mechanisms may change over time. The unresolved heterogeneity of PD may have contributed to the failure of past clinical trials, which attempted to slow the course of PD. We thus conclude that PD patients need personalized therapeutic approaches tailored to specific phenomenological and etiologic subtypes of disease.

## Introduction

A disease-modifying therapy that slows or prevents progression is the single most important unmet need in the treatment of Parkinson’s disease (PD). However, it is increasingly recognized that there is no PD as such, but the fundamental parkinsonian features of what we call PD occur in a number of more or less distinct conditions (Obeso 2017). Of note, the sporadic form, which is widely denominated as idiopathic PD also constitutes a syndrome comprising different subtypes and likely different etiologies. Among the presumed mechanisms, the contributions of inflammatory processes, glia cells, intestinal microbiota and the immunological status to the evolution of the disease remain largely unexplored (Ahmed et al. 2017; Bedarf 2017; De Miranda et al. 2022; Metta et al. 2022; Liu et al. 2021). Aggregation and (presumed) spreading of alpha-synuclein (*α*-syn) on the other hand clearly seem to constitute a critical event in PD pathophysiology and the recent failure of different monoclonal antibodies directed against aggregated *α*-syn in two therapeutic trails left the scientific community somewhat puzzled (Whone 2022).

It is thus crucially important to consider the heterogeneity of pathways and mechanisms to get closer to the different causes of neuronal demise and to define targets for a given population of PD patients. Importantly, in the course of the disease over decades, targets may change over time. Brundin and colleagues proposed a three-stage model of disease progression, each driven by different factors (triggers, facilitators and aggravators and pointed out, that molecular mechanisms triggering the initial phases of the disease may be different from later stages (Johnson et al. 2019). In this model, apart from causative gene mutations that result in functionally impaired proteins, triggers encompass environmental factors, such as pathogens, toxins or trauma. Disease mechanisms initiated by these triggers are perpetuated by individual facilitators or aggravators, i.e. mitochondrial dysfunction or systemic inflammatory reactions could contribute to disease progression; impaired autophagy and neuroinflammatory events may play a role in propagation and accelerated dysfunction. Following this concept, it appears plausible that inflammatory reactions as an example may constitute a drugable target only in a particular, probably early time-window and the same may hold true for aggregation of *α*-syn and other presumed targets in PD pathophysiology. Future therapies therefore not only need to consider the individual geno and molecular phenotype, but also the disease type and stage of individual patients.

### Clinical phenotypes, pathological findings and genetic predispositions

While the initial disease concepts assumed a rather uniform clinical course of PD following the loss of dopaminergic innervation, recent cohort studies have confirmed that PD indeed exhibits high phenotypic variability (Bartl et al. 2022). Independent analyses suggest four main clinical phenotypes, which at least in part seem to coincide with clinico-pathological findings: patients with non-tremor-dominant postural instability and gait dominated PD (PIGD subtype) display more cortical Lewy bodies (LB) and amyloid β plaques compared with tremor dominant PD patients (Selikhova et al. 2009). Beyond the clinically obvious motor deterioration distinguishing these subtypes, the extensive spectrum of non-motor symptoms (NMS), particularly cognitive, affective, sleep-related, and autonomic dysfunction represents clinical challenges and may define additional subtypes. (Weintraub et al. 2022; Bloem et al. 2021). Among the NMS of PD, dementia in the course of the disease (PDD), can lead to loss of everyday functioning, shortening of life expectancy due to faster progression and a significantly higher burden on family caregivers (Koros et al. 2022). Unfortunately, the prognoses associated with these subtypes are inconsistent and over time a shift from prognostically favorable phenotype to a more unfavorable subtype may occur—a phenomenon which could be explained by different individual predispositions or aggravators as outlined above (Lee et al. 2019; Mohl et al. 2017; Coelln et al. 2021).

Similar to the clinical phenotypes, numerous extrastriatal pathologies have been described in recent years as pathophysiological correlates for NMS in addition to the known nigrostriatal dopaminergic denervation. With regard to the nervous system these include, but are not limited to cholinergic, serotonergic and noradrenergic pathways. The tremendous beneficial effects of dopamine replacement therapy certainly contributed to the initial focus on particular issues of dopamine metabolism and the vulnerability of dopaminergic neurons, but may have limited “out of the box “research efforts in the past. The notion that PD is by no means restricted to the substantia nigra pars compacta (SNpc) was corroborated and substantially advanced by Braak and co-workers’ findings and the hypothesis of an “ascending” pathology of Lewy bodies (LB). However, is not clear how LB pathology evolves over time in a given individual and not all patients brains display patterns of LB pathology, which comply with Braak’s staging: there is no dose-dependent correlation between Lewy pathology and cell loss or clinical features (Surmeier et al. 2017; Beach et al. 2021).

Although LB are considered hallmarks of synucleinopathies, including PD, there is widespread histological heterogeneity with regard to their distribution. The correlation between LB pathology and neuronal loss is far from clear as is the additional role of amyloid plaques which have been shown to be associated with cognitive dysfunction in PDD and DLB (Halliday et al. 2011). Recently harmonized consensus criteria employed the preferential distribution of LB pathology in the neocortex, the limbic system, or the brainstem in correlation with the clinical PD or PDD presentation (Attems et al. 2021).

Mutations in different combinations can produce different phenotypes with different rates of progression (Iwaki et al. 2019). In addition to a specific PD susceptibility, concomitant diseases may also contribute to the observed heterogenous patterns of symptoms and progression rates with implication for therapeutic approaches (Liu et al. 2021). A D2 receptor polymorphism can co-determine the response to rasagiline and an α-syn polymorphism or a glucocerebrosidase (GBA) mutation can co-determine the efficacy of deep brain stimulation (Krüger et al. 2017; Masellis et al. 2016; Ligaard et al. 2019). If we assume that the degenerative process is indeed initiated at a given timepoint in an individual’s lifetime by a trigger, it is conceivable that genetic variability and exogenous factors (facilitators and aggravators) underlie the inter-individual differences with regard to the spectrum of symptoms and their progression.

Recent advances in genetics, in particular the conduction of “genome-wide association studies” (GWAS) in ever larger and more diverse populations of patients (Nalls et al. 2019) and controls have allowed a deeper insight into the genetic underpinnings of apparently sporadic PD. These studies suggest that overall heritability of PD accounts for 25–30%. From these data, so-called polygenic risk scores (PRS) can be constructed that can help predict an individual’s risk of developing PD. PRS are calculated by combining information from multiple genetic variants that have been found to be associated with a particular trait or disease in a GWAS. In the case of PD, hundreds of genetic variants have been identified that contribute to disease risk. To construct a PRS, a lead single nucleotide polymorphism (SNP) from each associated region is assigned a “weight” based on its effect size as determined by the association study. This information is then combined into a single score to predict an individual’s risk for PD.

In addition to predicting an individual's disease risk, PRS can theoretically also be used to study the relative contribution of different cellular pathways and function to disease pathogenesis in a given individual. For example, PRS can be constructed exclusively from variants linked to one of several incriminated cellular pathways, like the lysosomal-endosomal protein degradation pathway or mitochondrial maintenance and quality control. However, the potential of such studies is still limited, as in most cases, the causative genes and variants in a genomic region associated with an altered risk for PD are still unknown, i.e. only a very limited number of genes and loci can be reliably included at this point. More research is needed to further dissect the high phenotypic variability of PD and to define reliable subtypes, based on clinical phenotyping, biomarkers, or -omics findings. In addition to canonical genetics, epigenetic changes will have to be considered as well (Wüllner et al. 2016). Several approaches are being explored in observational cohorts, including follow-up of cohorts with certain prodromal features such as hyposmia, rapid eye movement sleep behavior disorders, or IPD gene carriers (Mahlknecht et al. 2022; Marini et al. 2020), but, as of yet, no clear-cut approach has emerged.

### Pathophysiological concepts, pathways and mechanisms

#### ɑ-synuclein and Lewy bodies

ɑ-syn pathology is not specific for sporadic PD but is found also in Multiple Systems Atrophy (MSA) and Alzheimer Disease (AD) (Kaufmann and Goldstein 2010; Jellinger 2020). In contrast, there is no evidence for the generation of LB in postencephalitic Parkinsonism (Cadar et al. 2021; Ling et al. 2016) and certain familial forms of PD like PINK 1 autosomal recessive early PD (Takanashi et al. 2016) and some cases of LRRK2 (PARK8) late onset PD (Pont-Sunyer et al. 2017). Thus, LB pathology not necessarily coincides with clinical Parkinsonism. Neurodegeneration of the SNpc might precede LB pathology and whether LB pathology correlates to dopaminergic cell loss in both, the SN and the striatum has been questioned (Beach et al. 2021; Parkkinen et al. 2011). In addition, only 30% of patients with a neurodegeneration disorder were diagnosed with ɑ-syn positive dorsal nuclei of the vagus, SN and/or basal forebrain nuclei (Parkkinen et al. 2005). In another study the dorsal nucleus of the vagus was preserved in about 7–16% of PD (Jellinger 2019 for review). Mori et al. (2006) studied the relationship between accumulation of ɑ-syn and tyrosine hydroxylase (TH) immunoreactivity. These authors showed a close relationship of ɑ-syn accumulation, and loss of both TH-IR and neurons. 10% of pigmented neurons in the SN and 54.9% of those in the LC contained abnormal ɑ-synuclein aggregates. Furthermore, 82.3% of pigmented neurons bearing ɑ-syn aggregates in the SN and 39.2% of those in LC lacked TH-IR (Mori et al. 2006). The heterogeneity of sporadic PD presumably also includes a variable participation of *ɑ*-syn at the pathological basis. To the best of our knowledge, LB are mature aggresomes, and therefore per se constitute a protective response of neurons to the accumulation of *α*-syn aggregates and numerous other proteins and lipids, i.e. mitochondrial membrane debris in particular. Aggresomes are formed at the microtubule-organizing center whenever transport of aggregates supersedes autophagic degradation (Kopito 2000). The process of aggresome maturation and LB formation involves posttranslational modifications and interactions with membranous intracellular structures, and does entail a series of functional cellular deficits. Consequently, LB cannot be considered unequivocally protective (Mahul-Mellier et al. 2020).

Interestingly, experimental studies using MPTP demonstrated LB-like aggregations only after long-term MPTP application but not after acute MPTP intoxication (Burns et al. 1984; Meredith and Rademaker 2011), suggesting that *α*-syn pathology in sporadic PD could represent a secondary phenomenon, triggered by aberrant metabolic processes, which had been induced earlier. In addition to mitochondrial dysfunction, the most recent implication of *α*-syn in immune responses is interesting. Upregulation of *α*-syn following immune activation has been suggested as a possible trigger of PD, after earlier findings of Beckham and co-workers, showing that *α*-syn expression restricts RNA viral infections in the brain (Kasen et al. 2022; Beatman et al. 2015).

#### Misfolding, aggregation and cellular quality control mechanisms

Misfolding and aggregation of proteins are common phenomena in neurodegeneration and both a propensity to aggregate in the first place and reduced capacity to discard misfolded proteins properly are important. Genetic deficits in either systems can trigger PD, as can multiplications or mutations of *α*-syn, which increase the likelihood of formation of toxic fibrils. The complex issue, whether *α*-syn takes up a soluble tetrameric form in the first place and that conversion into monomeric *α*-syn constitutes the first step towards oligomeric intermediates and eventually fibrillar aggregates has not been finally resolved (Nuber et al. 2018). The cellular quality control mechanisms include the ubiquitin–proteasome system and the auto-lysosomal system. Heterozygous (recessive) mutations in the lysosomal enzyme Glucocerebrosidase (GBA) gene constitute the most common genetic predisposition towards PD. Several steps are involved in aggregate clearance, starting with the recognition of misfolded proteins by ubiquitin ligases. Ubiquitinated monomers are degraded by the proteasome whereas larger accumulations of misfolded proteins require autophagy for degradation. In autophagy, ubiquitinated proteins are recognized by adaptor proteins, like p62 which interact with pre-autophagosomal membranes by binding to LC3. The autophagic membrane subsequently engulfs the aggregate and degradation occurs by fusion with lysosomes. In most cells, small aggregates are bound to dynein motor proteins through a second set of adaptor proteins, and thereby concentrated at the microtubule-organizing center. The subsequent steps of auto-lysosomal degradation are compartmentalized particularly in neurons: fusion to lysosomes occurs in the soma only. Autophagosomes formed in the distal axon need to be transported for degradation, which explains the presence of axonal *α*-syn deposits and the importance of transport deficits for PD pathogenesis.

#### The gut–brain axis and subtypes of disease

The peripheral autonomic nervous system is uniquely involved in LB disorders. Aggregated *α*-syn have been found in the enteric nervous system up to 20 years before diagnosis (Stokholm et al. 2016). The dual-hit hypothesis proposes that *α*-syn aggregation is triggered in the enteric nervous system and that it then spreads via the vagus nerve to the dorsal motor nucleus in all cases of PD (Hawkes et al. 2007). In support, two epidemiology studies showed that full truncal vagotomy in humans reduced subsequent risk of PD by 4050% (Svensson et al. 2015; Liu et al. 2017). Animal studies confirm that gut-injected *α*-syn seeds leads to spreading of *α*-syn pathology and neurodegeneration in spatio-temporal patterns, which parallel the evolution of human PD (Berge et al. 2019; Kim et al. 2019).

It has been speculated that microbiome factors could be involved in initiating the first *α*-syn aggregation. The microbiome is altered in PD at the prodromal stage (Heinzel et al. 2021). Certain bacteria, which are commonly present in the gut microbiome, can produce amyloid proteins such as curli. These proteins can initiate enteric *α*-syn aggregation in animal models (Chen et al. 2016). Several studies have shown that PD patients show signs of leaky gut syndrome, which could facilitate that detrimental microbiome-derived trigger factors, such as curli, can get into contact with the enteric nervous system (Forsyth et al. 2011). Inflammatory bowel disorders also increase the risk of subsequent PD, which may suggest that inflammation of many different types and origins may promote *α*-syn aggregation and therefore increase the risk of PD (Peter el al. 2018; Villumsen et al. 2019). However, disentangling the cause and effect of gut–brain axis factors in PD is complicated by the extended prodromal phase, which can span more than 10–20 years (Savica et al. 2010). For instance, it is possible that leaky gut syndrome and alterations in the microbiome may be a secondary cause of PD, and not an upstream trigger of *α*-syn aggregation.

Recently, it has been hypothesized that LB disorders, including PD, DLB, iRBD, and PAF, can be divided according to a body-first and brain-first dichotomy (Borghammer et al. 2021; Horsager et al. 2020). In body-first patients, LB pathology is triggered in the gut and spreads via the vagus and sympathetic spreading route to sympathetic ganglia and trunk. Such patients therefore develop autonomic symptoms and neurodegeneration, and RBD before Parkinsonism emerges. In brain-first patients, LB pathology is triggered in the olfactory bulb and/or amygdala and reaches the SN very rapidly. These patients therefore have a shorter prodromal phase and few or no non-motor symptoms before diagnosis. This disease model is supported by clinical imaging studies, which shows that patients who developed RBD years before diagnosis, show marked loss of sympathetic cardiac denervation and parasympathetic cholinergic innervation of the gut years before the SN starts to degenerate. In contrast, de novo PD patients, who are RBD-negative at diagnosis, generally show normal or near-normal sympathetic and parasympathetic innervation of peripheral organs, but marked nigrostriatal denervation (Knudsen et al. 2018; Horsager et al. 2020; Nishikawa et al. 2022; Kim et al. 2017). This dichotomy is supported by brain bank studies, which have shown that the large majority of cases with very early incidental LB disease can be categorized into two types. One group has pathology in the amygdala and olfactory bulb, but no pathology in the lower brainstem or autonomic system. The other group has pathology in the autonomic systems and lower brainstem but little or no pathology in the amygdala and olfactory bulb (Tanei et al. 2021; Raunio et al. 2019; Borghammer et al. 2021, 2022).

LB disorders are complex and heterogeneous, so a dichotomous system is potentially an oversimplification, which was recently pointed out (Fearon et al. 2021; Borghammer and Horsager 2021). Yet, the body-first vs. brain-first model is based on an assumption, which makes this particular dichotomy logically consistent. It is proposed that LB pathology in most patients starts in a single location, perhaps inside a single neuron, and then spreads from there. Since the nervous system by definition has two main compartments, the peripheral and the central, it follows that the first pathology will arise in either the peripheral compartment (gut) or the central compartment (olfactory bulb or amygdala). Thus, whether or not this disease model is an oversimplification is dependent on the veracity of the underlying assumption of a single-location origin.

Interestingly, the idea of a single-location origin allows the body- vs. brain-first model to explain why some patients with LB disease have asymmetric Parkinsonism (Borghammer 2021). The connectome in mammalian brains is highly lateralized. Ipsilateral connections outnumber contra-lateral, commissural connections 100:1. Thus, if LB pathology arises in one olfactory bulb and spreads proportional to connection strength, it will lead to degeneration in the ipsilateral SN first. Thus, brain-first patients will generally show asymmetric dopamine loss. However, the vagus and sympathetic innervation of the gut show left–right overlap in innervation patterns. Thus, a single origin site in the gut leads to more symmetric propagation through the left and right vagus, simultaneously. In body-first patients, the SN is therefore affected in a more symmetric fashion and such patients should therefore have more symmetric dopamine loss on imaging. These patterns of asymmetric vs. asymmetric dopamine loss in brain first vs. body-first patients, respectively, fits well with in vivo clinical imaging data (Knudsen et al. 2021; Walker et al. 2004; Cao et al. 2020).

#### Oxidative stress, mitochondria, iron and melanin

Oxidative stress, i.e. a dysbalance between the production of reactive oxygen species (ROS) and the biological system's ability to detoxify the reactive intermediates has been implicated in the progression of PD and other neurodegenerative diseases, in particular AD and Motor Neuron Disease (MND). In PD reduced glutathione (GSH) in substantia nigra tissue was identified 30 years ago and sparked multiple lines of research into mechanisms of ROS balance (Sian et al. 1994). We and others have shown that depletion of GSH in animal models of PD renders dopaminergic neurons of the substantia nigra more vulnerable and that a chronic loss of GSH has severe consequences for mitochondrial function (Wüllner et al. 1999). Interestingly, these findings implicate astrocytes, which are indispensable for GSH production and supply to neurons into the pathophysiological concept of PD. On the other hand, subsequent experiments revealed, that loss of GSH alone is not responsible for nigrostriatal damage in PD. Rather, GSH depletion may enhance the susceptibility of substantia nigra neurons to destruction by endogenous or exogenous toxins (Toffa et al. 1997).

Oxidative stress is further enhanced by the metabolites of dopamine. Under physiological conditions cytoplasmic dopamine in part is metabolized by monoamine oxidase in the outer mitochondrial membrane to form 3,4-dihydroxyphenylacetaldehyde (DOPAL). DOPAL via aldehyde dehydroxygenase (ALDH) is converted to 3,4-dihydroxyphenylacetic acid (DOPAC). In PD, ALDH A1A is nearly absent and it has been suggested that an increase of DOPAL increases the risk neurodegenerative processes (Goldstein et al. 2013). DOPAL is more than 1000 times as potent as dopamine to induce mitochondrial damage. Hydrogen peroxide, another metabolite of oxidative deamination processes, exerts toxicity via reactions with DOPAL to form hydroxyl radicals and reacts with iron, increasing the iron-induced oxidative stress (Goldstein et al. 2013). Hydroxyl radicals peroxidate lipid membranes and the lipid peroxidation product 4-hydroxynonenal inhibits ALDH, leading to further accumulation of DOPAL. Both, DOPAL and iron-induced oxidative stress are prominent in their reaction with ɑ-syn. DOPAL has been reported to potently oligomerize ɑ-syn and iron-induced oxidative stress might oxidase the 4-tyrosine rests of ɑ-synuclein, so that structural changes of the molecule hinder any proteasomal degradation (Burke et al. 2008; Riederer et al. 2021). Inhibition of monoamine oxidase therefore represents a valuable treatment option to decrease both DOPAL- and hydrogen peroxide formation and to reduce the burden of ROS leading to neurodegeneration (Naoi et al. 2020).

Iron and neuromelanin (NM) show a significant increase in the SNpc with age; aging is a major risk factor for PD and iron is significantly increased in the SNpc of PD patients (Foley et al. 2022, for review). The role of NM as a trigger of PD is of current interest as ɑ-syn and NM are prominent hallmarks in the pathology of PD and ɑ-syn has been detected in NM isolated post mortem from PD SN (Tribl et al. 2005). NM exerts protective action by quenching transition metals, xenobiotics, lipids and various proteins but also contributes to degenerative processes under special intraneuronal conditions (Moreno-Garcia et al. 2021). The binding capacity of NM is limited and changes in the composition of the cytoplasmic fluid may release iron from NM, causing an increase of redox-active iron and iron-induced oxidative stress. NM increases with age and NM containing catecholaminergic neurons of the SN and the locus coeruleus are particularly vulnerable in PD (Cai et al. 2023). It is assumed that NM (which is absent in the SN of rats) could reduce the toxicity of iron. The importance of mitochondrial function and impairment for ROS generation and the pathophysiology of PD has been extensivly reviewed in several excellent recent publications (for review: Rehman et al. 2023) and has also been linked to important steps in neuroinflammation (Han and Le 2023; Magalhães, Cardoso 2023).

As of yet however, clinical trials of antioxidants failed to prove efficacious in neurodegenerative diseases and a recent clinical study of the iron-chelator deferiprone even led to worsening of the verum treated PD patients (Devos 2020) and again the question arises, whether the inability to show disease modification in PD is due to preclinical research providing misleading encouragement.

#### Insulin and incretin signaling pathways

Epidemiological studies suggest that diabetes and hyperglycemia are associated with an increased incidence and severity of PD. Early case–control studies suggested a decreased risk for type 2 diabetes mellitus (T2DM) to develop PD (Powers et al. 2006). However, larger and more recent cohort studies have unequivocally shown that T2DM is associated with an increased risk of developing PD (Hu et al. 2007). A current meta-analysis including all above mentioned studies revealed an overall effect estimate with 95% confidence interval of 1.21 (1.07, 1.36) (Chohan et al. 2021). Diabetes was associated with motor and cognitive progression in PD patients. In addition to diabetes, increased glycated hemoglobin (HbA1c) was associated with an unfavorable motor outcome in different PD cohorts (Zittel et al. 2021). Unsurprisingly, prevalent diabetes and high HbA1c levels were both linked with increased neuroaxonal damage quantified by neurofilament light chain (NfL) levels (Uyar et al. 2022). Different mechanisms might underlie the increased neurodegeneration and aggravated PD pathology. DM has been associated with lower striatal dopamine transporter binding and increased tau pathology, both in patients with diabetes and w/o PD (Pagano et al. 2018). Altered glucose homeostasis could lead to mitochondrial dysfunction, increased endoplasmic reticulum (ER) stress, inflammatory processes and dysregulated protein degradation. Concerning PD specific mechanisms, insulin resistance and hyperglycemia can decrease dopamine levels and release, lead to dopaminergic dysfunction and decrease striatal dopamine turn-over (Montefusco et al. 1983). Among anti-diabetic drugs, most studies have shown neuroprotective effects of metformin and especially glucagon-like-peptide 1 (GLP-1) agonists. In different PD mouse models, metformin attenuated the degeneration of the substantia nigra and improved motor deficits, probably through a positive effect on autophagy (Lu et al. 2016). In the last decade research has focused on the GLP-1 pathway as a distinct therapeutic target in PD.

GLP-1 is an endogenous hormone secreted from intestinal cells and amplifies the insulin release upon food intake. GLP-1 binds to respective receptors which are expressed in several organs like gut, heart, lung, kidney and brain. GLP-1 signaling can increase neurogenesis, reduce apoptosis, protect neurons from oxidative stress and reduce neuroinflammation (Chen et al. 2023 for review). Subsequently, GLP-1 is enzymatically degraded by dipeptidy peptidase-4 (DPP-4). Consequently, GLP-1 mimetics (i.e. GLP‑1 agonists) and enhancers (i.e. DPP-4 inhibitors) have been developed and approved for use in T2DM patients. Interestingly, a population-based cohort study revealed that PD incidence in patients with diabetes might vary according to their anti-diabetic treatment and that especially the use of GLP‑1 agonists and/or DPP-4 inhibitors are associated with a lower PD incidence (Brauer et al. 2020). Among approved GLP-1 agonists, exenatide has emerged as a promising disease-modifying drug in PD (Athauda et al. 2017). In a randomized, double-blind, placebo controlled trial, patients with moderate stage PD treated with exenatide 2 mg once weekly for 48 weeks had an Movement Disorders Society Unified Parkinson’s Disease Rating Scale (MDSUPDRS) part 3 score of 3.5 points less compared with the placebo group (28781108). Recruitment for the corresponding phase 3 trial with exenatide over a period of 2 years has been completed and results are expected in 2024. Clinical trials evaluating GLP-1 signaling are increasing (McFarthing et al. 2022). The clinical trial database reveals in addition to studies with exenatide lists studies with other GLP-1 agonists (liraglutide and semaglutide), which are or are going to be evaluated in PD patients. Similar to exenatide, liraglutide and semaglutide conferred protective effects in rodent PD models with 6-hydroxydopamine (6OHDA), 1-Methyl-4-phenyl-1,2,3,6-tetrahydropyridine (MPTP) and human A53T α-synuclein transgenic mice (Zhang et al. 2019). Despite its astonishing effects in preclinical studies and clinical trials, the underlying protective mechanisms of GLP-1 in PD remains unclear at the moment.

As the insulin/Insulin receptor pathway plays a major role in the etiopathogenesis of cognitive decline and particular in AD it has been speculated that disturbance of this pathway is of particular importance in PDD (Salkovic-Petrisic et al. 2013).

#### T-cell autoimmunity

T-cell autoimmunity constitutes the most recent addition to the collection of factors influencing—and potentially triggering—a-syn pathology and PD. In a seminal paper, Sulzer and colleagues found that a-syn epitopes are displayed by the major histocompatibility complex and initiate a T-cell response in patients with PD (Sulzer et al. 2017). Specifically, IL-17 producing T lymphocytes mediated neuronal cell death in a combined model of patient and stem cell derived neurons with autologous T cells. Collectively, these findings indicate that the T-cell response might contribute to pathogenesis in PD. Importantly, this offers targets for protective interventions, like the FDA-approved anti-IL-17 antibody, secukinumab (Sommer et al. 2018).

#### Resilience

One of the most intriguing questions is why neurodegenerative diseases, such as PD, occur largely in an aging population. The contribution of the natural aging process to neurodegeneration and the mechanisms which are lost over time that confer resilience to degeneration at younger age are unknown. Remarkably though, sex seems to confer neuroprotection: many neurodegenerative diseases, including PD, have a male predominance and it is likely that female individuals carry increased resilience (Moisan et al. 2016). Evidence from C. elegans suggests that the knockout of specific microRNA (mir-2) can attenuate α-syn neurotoxicity, suggesting that it’s molecular targets could act as neuroprotective modulators (Gaeta et al. 2022). In Drosophila, sex- and age-related differences in vulnerability of dopaminergic (DAergic) neurons could be related to the expression of the vesicular glutamate transporter (VGLUT). Male Drosophila show a stronger loss of DAergic neurons with age compared to females. Interestingly, females have higher levels of VGLUT expression in DAergic neurons, which is also true for humans. Resilience in DAergic neurons could thus be modulated by VGLUT, which could also represent an interesting therapeutic strategy (Buck et al. 2021). In C57BL/6 mice, DAergic neuron firing decreases with age in males, whereas it is not affected in females (Howell et al. 2020). Interestingly, expression of PARK2 increased in males, which could contribute to this selective vulnerability. Not all findings observed in short-lived animals, such as C. elegans, Drosophila or mice can be translated to humans. Nevertheless, these molecular examples showcase the possibility to not only interact with pathology itself, but also exploit protective mechanisms to modulate neurodegeneration.

## Conclusions

The discussion of the heterogeneity of symptoms and pathophysiological mechanisms is a re-occuring, well-known topic not only in PD and other neurological conditions. Many other medical conditions have attracted “splitters and lumpers” for different, albeit well taken reasons (Espay et al. 2020).

A particular line of thought may be worth to be (re-)considered (again). The exploration of the mechanisms implicated in the various cascades of neurodegeneration outlined above may have led “off track” and we might have missed an “elephant in the room”: the exogenous infectious agents which might trigger sporadic PD in susceptible individuals upon entry via the olfactory bulb (brain first) or the gut (body-first) (Borghammer et al. 2021; Horsager et al. 2020). Viral and microbial agents have been reported to produce molecular hallmarks of neurodegeneration, such as the deposit of misfolded protein aggregates, oxidative stress, deficient autophagic processes and synaptopathies (De Chiara et al 2012). The activation of inflammatory processes and host immune responses causes chronic damage resulting in alterations of neuronal function and viability. Midbrain dopamine neurons are believed to be particularly susceptible to inflammation and recent biomarker studies indeed support an ongoing systemic inflammation in PD (Johnson et al. 2019; Yacoubian et al. 2023).

Convincing experimental evidence for a post-infectious cascade of events was provided as early as 2009, when Jang and Co-workers demonstrated that H5N1 influenza virus can enter the central nervous system and induce neuroinflammation and neurodegeneration (Jang et al. 2009). They showed that the virus traveled from the peripheral nervous system into the CNS to higher levels of the neuroaxis in line with Braak’s hypothesis of an ascending progression of pathology. In regions infected by H5N1 virus, activation of microglia, α-syn phosphorylation and aggregation persisted after resolution of the acute infection and a significant loss of dopaminergic neurons in the SNpc was noted after infection.

The currently available evidence on viral-induced Parkinsonism with a focus on potential pathophysiological mechanisms and clinical features and the evidence of viral infections as a risk factor for developing PD has recently been reviewed by Chaudhuri and co-workers (Leta et al. 2022). It is conceivable that particular agents (among them probably neurotropic viruses) could initiate neurodegenerative disorders of protein aggregation, including PD. Very recent findings even pointed to upregulation of α-syn following immune activation, suggesting that similar to what is being discussed in multiple sclerosis, a viral infection might be a necessary but not necessarily a sufficient insult for the initiation of PD (Kasen et al. 2022). Recently, this discussion was fueled by the COVID-19 pandemic and the question has been raised whether SARS-CoV-2 could be a trigger for neurodegeneration (reviewed in Lingor et al. 2022). The above outlined heterogeneity including the various pathophysiological pathways of sporadic PD in the course of disease might thus reflect the individuals’ specific predisposition and immunologic reactions towards the initial culprit.

## Data Availability

No data have been displayed in the narrrative review.

## References

[CR1] Ahmed H, Abushouk AI, Gabr M, Negida A, Abdel-Daim MM (2017). Parkinson’s disease and pesticides: a meta-analysis of disease connection and genetic alterations. Biomed Pharmacother.

[CR2] Athauda D, Maclagan K, Skene SS, Bajwa-Joseph M, Letchford D, Chowdhury K, Hibbert S, Budnik N, Zampedri L, Dickson J, Li Y, Aviles-Olmos I, Warner TT, Limousin P, Lees AJ, Greig NH, Tebbs S, Foltynie T (2017). Exenatide once weekly versus placebo in Parkinson's disease: a randomised, double-blind, placebo-controlled trial. Lancet.

[CR3] Attems J, Toledo JB, Walker L, Gelpi E, Gentleman S, Halliday G, Hortobagyi T, Jellinger K, Kovacs GG, Lee EB, Love S, McAleese KE, Nelson PT, Neumann M, Parkkinen L, Polvikoski T, Sikorska B, Smith C, Grinberg LT, Thal DR, Trojanowski JQ, McKeith IG (2021). Neuropathological consensus criteria for the evaluation of Lewy pathology in post-mortem brains: a multi-centre study. Acta Neuropathol.

[CR4] Bartl M, Dakna M, Schade S (2022). Longitudinal change and progression indicators using the movement disorder society-unified Parkinson's disease rating scale in two independent cohorts with early Parkinson's disease. J Parkinsons Dis.

[CR5] Beach TG, Adler CH, Sue LI, Shill HA, Driver-Dunckley E, Mehta SH, Intorcia AJ, Glass MJ, Walker JE, Arce R, Nelson CM, Serrano GE (2021). Vagus nerve and stomach synucleinopathy in Parkinson's disease, incidental lewy body disease, and normal elderly subjects: evidence against the "body-first" hypothesis. J Parkinsons Dis.

[CR6] Beatman EL, Massey A, Shives KD, Burrack KS, Chamanian M, Morrison TE, Beckham JD (2015). alpha-synuclein expression restricts RNA viral infections in the brain. J Virol.

[CR7] Bedarf JR, Hildebrand F, Coelho LP, et al (2017) Functional implications of microbial and viral gut metagenome changes in early stage L-DOPA-naïve Parkinson's disease patients. Genome Med 28;9(1):39. 10.1186/s13073-017-0428-y 10.1186/s13073-017-0428-y PMC5408370 28449715

[CR8] Bloem BR, Okun MS, Klein C (2021). Parkinson's disease. Lancet.

[CR9] Borghammer P (2021). The α-synuclein origin and connectome model (SOC Model) of Parkinson's disease: explaining motor asymmetry, non-motor phenotypes, and cognitive decline. J Parkinsons Dis.

[CR10] Borghammer P, Horsager J, Andersen K, Van Den Berge N, Raunio A, Murayama S, Parkkinen L, Myllykangas L (2021). Neuropathological evidence of body-first vs. brain-first Lewy body disease. Neurobiol Dis.

[CR11] Borghammer P, Just MK, Horsager J, Skjærbæk C, Raunio A, Kok EH, Savola S, Murayama S, Saito Y, Myllykangas L, Van Den Berge N (2022). A postmortem study suggests a revision of the dual-hit hypothesis of Parkinson's disease. NPJ Parkinsons Dis.

[CR12] Brauer R, Wei L, Ma T, Athauda D, Girges C, Vijiaratnam N, Auld G, Whittlesea C, Wong I, Foltynie T (2020). Diabetes medications and risk of Parkinson's disease: a cohort study of patients with diabetes. Brain.

[CR13] Buck SA, Steinkellner T, Aslanoglou D, Villeneuve M, Bhatte SH, Childers VC, Rubin SA, De Miranda BR, O'Leary EI, Neureiter EG, Fogle KJ, Palladino MJ, Logan RW, Glausier JR, Fish KN, Lewis DA, Greenamyre JT, McCabe BD, Cheetham CEJ, Hnasko TS, Freyberg Z (2021). Vesicular glutamate transporter modulates sex differences in dopamine neuron vulnerability to age-related neurodegeneration. Aging Cell.

[CR14] Burke WJ, Kumar VB, Pandey N, Panneton WM, Gan Q, Franko MW, O'Dell M, Li SW, Pan Y, Chung HD, Galvin JE (2008). Aggregation of alpha-synuclein by DOPAL, the monoamine oxidase metabolite of dopamine. Acta Neuropathol.

[CR15] Burns RS, Markey SP, Phillips JM, Chiueh CC (1984). The neurotoxicity of 1-methyl-4-phenyl-1,2,3,6 tetrahydropyridine in the monkey and man. Can J Neurol Sci.

[CR16] Cadar D, Jellinger KA, Riederer P, Strobel S, Monoranu CM, Tappe D (2021). No metagenomic evidence of causative viral pathogens in postencephalitic Parkinsonism following encephalitis lethargica. Microorganisms.

[CR17] Cai W, Wakamatsu K, Zucca FA, Wang Q, Yang K, Mohamadzadehonarvar N, Srivastava P, Tanaka H, Holly G, Casella L, Ito S, Zecca L, Chen X (2023). DOPA pheomelanin is increased in nigral neuromelanin of Parkinson's disease. Prog Neurobiol.

[CR18] Cao R, Chen X, Xie C, Hu P, Wang K (2020). Serial dopamine transporter imaging of nigrostriatal function in Parkinson's disease with probable REM sleep behavior disorder. Front Neurosci.

[CR19] Chen SG, Stribinskis V, Rane MJ, Demuth DR, Gozal E, Roberts AM, Jagadapillai R, Liu R, Choe K, Shivakumar B, Son F, Jin S, Kerber R, Adame A, Masliah E, Friedland RP (2016). Exposure to the functional bacterial amyloid protein curli enhances alpha-synuclein aggregation in aged fischer 344 rats and *Caenorhabditis elegans*. Sci Rep.

[CR20] Chen SD, Chuang YC, Lin TK, Yang JL (2023). Alternative role of glucagon-like Peptide-1 receptor agonists in neurodegenerative diseases. Eur J Pharmacol.

[CR21] Chohan H, Senkevich K, Patel RK, Bestwick JP, Jacobs BM, Bandres Ciga S, Gan-Or Z, Noyce AJ (2021). Type 2 diabetes as a determinant of Parkinson's disease risk and progression. Mov Disord.

[CR22] De Chiara G, Marcocci ME, Sgarbanti R, Civitelli L, Ripoli C, Piacentini R, Garaci E, Grassi C, Palamara AT (2012). Infectious agents and neurodegeneration. Mol Neurobiol.

[CR23] De Miranda BR, Goldman SM (2022). Preventing Parkinson's disease: an environmental agenda. J Parkinsons Dis.

[CR24] Devos D, Labreuche J, Rascol O, Corvol JC, Duhamel A, Guyon Delannoy P, Poewe W, Compta Y, Pavese N, Růžička E, Dušek P, Post B, Bloem BR, Berg D, Maetzler W, Otto M, Habert MO, Lehericy S, Ferreira J, Dodel R, Tranchant C, Eusebio A, Thobois S, Marques AR, Meissner WG, Ory-Magne F, Walter U, de Bie RMA, Gago M, Vilas D, Kulisevsky J, Januario C, Coelho MVS, Behnke S, Worth P, Seppi K, Ouk T, Potey C, Leclercq C, Viard R, Kuchcinski G, Lopes R, Pruvo JP, Pigny P, Garçon G, Simonin O, Carpentier J, Rolland AS, Nyholm D, Scherfler C, Mangin JF, Chupin M, Bordet R, Dexter DT, Fradette C, Spino M, Tricta F, Ayton S, Bush AI, Devedjian JC, Duce JA, Cabantchik I, Defebvre L, Deplanque D, Moreau C; FAIRPARK-II Study Group (2020) Trial of Deferiprone in Parkinson's Disease. N Engl J Med 387(22):2045-2055.10.1056/NEJMoa2209254

[CR25] Espay AJ, Kalia LV, Gan-Or Z, Williams-Gray CH, Bedard PL, Rowe SM, Morgante F, Fasano A, Stecher B, Kauffman MA, Farrer MJ, Coffey CS, Schwarzschild MA, Sherer T, Postuma RB, Strafella AP, Singleton AB, Barker RA, Kieburtz K, Olanow CW, Lozano A, Kordower JH, Cedarbaum JM, Brundin P, Standaert DG, Lang AE (2020). Disease modification and biomarker development in Parkinson disease: revision or reconstruction?. Neurology.

[CR26] Fearon C, Lang AE, Espay AJ (2021). The logic and pitfalls of Parkinson's disease as "brain-first" versus "body-first" subtypes. Mov Disord.

[CR27] Foley PB, Hare DJ, Double KL (2022). A brief history of brain iron accumulation in Parkinson disease and related disorders. J Neural Transm (vienna).

[CR28] Forsyth CB, Shannon KM, Kordower JH, Voigt RM, Shaikh M, Jaglin JA, Estes JD, Dodiya HB, Keshavarzian A (2011) Increased intestinal permeability correlates with sigmoid mucosa alphasynuclein staining and endotoxin exposure markers in early Parkinson's disease. PLoS One 6(12):e28032. 10.1371/journal.pone.0028032. 10.1371/journal.pone.0028032 PMC3228722 22145021

[CR29] Gaeta AL, Nourse JB Jr, Willicott K, McKay LE, Keogh CM, Peter K, Russell SN, Hamamichi S, Berkowitz LA, Caldwell KA, Caldwell GA (2022) Systemic RNA interference defective (SID) genes modulate dopaminergic neurodegeneration in *C. elegans*. PLoS Genet 18(8):e1010115. 10.1371/journal.pgen.1010115 10.1371/journal.pgen.1010115 PMC9432717 35984862

[CR30] Goldstein DS, Sullivan P, Holmes C, Miller GW, Alter S, Strong R, Mash DC, Kopin IJ, Sharabi Y (2013). Determinants of buildup of the toxic dopamine metabolite DOPAL in Parkinson's disease. J Neurochem.

[CR31] Halliday GM, Holton JL, Revesz T, Dickson DW (2011). Neuropathology underlying clinical variability in patients with synucleinopathies. Acta Neuropathol.

[CR32] Han QQ, Le W (2023) NLRP3 inflammasome-mediated neuroinflammation and related mitochondrial impairment in Parkinson's disease. Neurosci Bull. 10.1007/s12264-023-01023-y. Epub ahead of print. 10.1007/s12264-023-01023-y PMC10169990 36757612

[CR33] Hawkes CH, Del Tredici K, Braak H (2007). Parkinson's disease: a dual-hit hypothesis. Neuropathol Appl Neurobiol.

[CR34] Heinzel S, Aho VTE, Suenkel U, von Thaler AK, Schulte C, Deuschle C, Paulin L, Hantunen S, Brockmann K, Eschweiler GW, Maetzler W, Berg D, Auvinen P, Scheperjans F (2021) Gut microbiome signatures of risk and prodromal markers of parkinson disease. Ann Neurol 90(3):E1E12. 10.1002/ana.26128 10.1002/ana.26128 34021620

[CR35] Horsager J, Andersen KB, Knudsen K, Skjærbæk C, Fedorova TD, Okkels N, Schaeffer E, Bonkat SK, Geday J, Otto M, Sommerauer M, Danielsen EH, Bech E, Kraft J, Munk OL, Hansen SD, Pavese N, Göder R, Brooks DJ, BergBorghammer D (2020). Brain-first versus body-first Parkinson's disease: a multimodal imaging case-control study. Brain.

[CR36] Howell RD, Dominguez-Lopez S, Ocañas SR, Freeman WM, Beckstead MJ (2020). Female mice are resilient to age-related decline of substantia nigra dopamine neuron firing parameters. Neurobiol Aging.

[CR37] Hu G, Jousilahti P, Bidel S, Antikainen R, Tuomilehto J (2007). Type 2 diabetes and the risk of Parkinson's disease. Diabetes Care.

[CR38] Iwaki H, Blauwendraat C, Leonard HL (2019). Genetic risk of Parkinson disease and progression: an analysis of 13 longitudinal cohorts. Neurol Genet.

[CR39] Jang H, Boltz D, Sturm-Ramirez K, Shepherd KR, Jiao Y, Webster R, Smeyne RJ (2009). Highly pathogenic H5N1 influenza virus can enter the central nervous system and induce neuroinflammation and neurodegeneration. Proc Natl Acad Sci USA.

[CR40] Jellinger KA (2019). Is Braak staging valid for all types of Parkinson's disease?. J Neural Transm (vienna).

[CR41] Jellinger KA (2020). Neuropathological assessment of the Alzheimer spectrum. J Neural Transm (vienna).

[CR42] Johnson ME, Stecher B, Labrie V, Brundin L, Brundin P (2019). Triggers, facilitators, and aggravators: redefining Parkinson's disease pathogenesis. Trends Neurosci.

[CR43] Kasen A, Houck C, Burmeister AR, Sha Q, Brundin L, Brundin P (2022). Upregulation of α-synuclein following immune activation: Possible trigger of Parkinson's disease. Neurobiol Dis.

[CR44] Kaufmann H, Goldstein DS (2010). Pure autonomic failure: a restricted Lewy body synucleinopathy or early Parkinson disease?. Neurology.

[CR45] Kim JS, Park HE, Park IS, Oh YS, Ryu DW, Song IU, Jung YA, Yoo IR, Choi HS, Lee PH, Lee KS (2017). Normal 'heart' in Parkinson's disease: is this a distinct clinical phenotype?. Eur J Neurol.

[CR46] Kim S, Kwon SH, Kam TI, Panicker N, Karuppagounder SS, Lee S, Lee JH, Kim WR, Kook M, Foss CA, Shen C, Lee H, Kulkarni S, Pasricha PJ, Lee G, Pomper MG, Dawson VL, Dawson TM, Ko HS (2019). Transneuronal propagation of pathologic α-synuclein from the gut to the brain models Parkinson's disease. Neuron.

[CR47] Knudsen K, Fedorova TD, Hansen AK, Sommerauer M, Otto M, Svendsen KB, Nahimi A, Stokholm MG, Pavese N, Beier CP, Brooks DJ, Borghammer P (2018). In-vivo staging of pathology in REM sleep behaviour disorder: a multimodality imaging case-control study. Lancet Neurol.

[CR48] Knudsen K, Fedorova TD, Horsager J, Andersen KB, Skjærbæk C, Berg D, Schaeffer E, Brooks DJ, Pavese N, Van Den Berge N, Borghammer P (2019) Asymmetric dopaminergic dysfunction in brainfirst versus body-first Parkinson's disease subtypes. J Parkinsons Dis 11(4):1677–1687. 10.3233/JPD-212761 10.3233/JPD-212761 34334424

[CR49] Kopito RR (2000). Aggresomes, inclusion bodies and protein aggregation. Trends Cell Biol.

[CR50] Koros C, Stefanis L, Scarmeas N (2022) Parkinsonism and dementia. J Neurol Sci 15;433:120015. 10.1016/j.jns.2021.120015 10.1016/j.jns.2021.120015 34642023

[CR51] Krüger R, Klucken J, Weiss D, Tönges L, Kolber P, Unterecker S, Lorrain M, Baas H, Müller T, Riederer P (2017). Classification of advanced stages of Parkinson's disease: translation into stratified treatments. J Neural Transm (vienna).

[CR52] Lee JW, Song YS, Kim H, Ku BD, Lee WW (2019). Alteration of tremor dominant and postural instability gait difficulty subtypes during the progression of Parkinson's disease: analysis of the PPMI cohort. Front Neurol.

[CR53] Leta V, Urso D, Batzu L, Lau YH, Mathew D, Boura I, Raeder V, Falup-Pecurariu C, van Wamelen D, Ray Chaudhuri K (2022). Viruses, parkinsonism and Parkinson's disease: the past, present and future. J Neural Transm (vienna).

[CR54] Ligaard J, Sannæs J, Pihlstrøm L (2019). Deep brain stimulation and genetic variability in Parkinson’s disease: a review of the literature. NPJ Parkinsons Dis.

[CR55] Ling H, Kearney S, Yip HL, Silveira-Moriyama L, Revesz T, Holton JL, Strand C, Davey K, Mok KY, Polke JM, Lees AJ (2016). Parkinson's disease without nigral degeneration: a pathological correlate of scans without evidence of dopaminergic deficit (SWEDD)?. J Neurol Neurosurg Psychiatry.

[CR56] Lingor P, Demleitner AF, Wolff AW, Feneberg E (2022). SARS-CoV-2 and neurodegenerative diseases: what we know and what we don't. J Neural Transm (vienna).

[CR57] Liu B, Fang F, Pedersen NL, Tillander A, Ludvigsson JF, Ekbom A, Svenningsson P, Chen H, Wirdefeldt K (2017). Vagotomy and Parkinson disease: a Swedish register-based matched-cohort study. Neurology.

[CR58] Liu G, Peng J, Liao Z et al (2021) International genetics of Parkinson disease progression (IGPP) consortium, Scherzer CR. Genome-wide survival study identifies a novel synaptic locus and polygenic score for cognitive progression in Parkinson's disease. Nat Genet 53(6):787–793. 10.1038/s41588-021-00847-6 10.1038/s41588-021-00847-6 PMC8459648 33958783

[CR59] Lu M, Su C, Qiao C, Bian Y, Ding J, Hu G (2016) Metformin prevents dopaminergic neuron death in MPTP/P-induced mouse model of Parkinson's disease via autophagy and mitochondrial ROS clearance. Int J Neuropsychopharmacol 19(9):pyw047. 10.1093/ijnp/pyw047 10.1093/ijnp/pyw047 PMC5043649 27207919

[CR60] Magalhães JD, Cardoso SM (2023). Mitochondrial signaling on innate immunity activation in Parkinson disease. Curr Opin Neurobiol.

[CR61] Mahlknecht P, Marini K, Werkmann M, Poewe W, Seppi K (2022). Prodromal Parkinson's disease: hype or hope for disease-modification trials?. Transl Neurodegener.

[CR62] Mahul-Mellier AL, Burtscher J, Maharjan N, Weerens L, Croisier M, Kuttler F, Leleu M, Knott GW, Lashuel HA (2020). The process of Lewy body formation, rather than simply α-synuclein fibrillization, is one of the major drivers of neurodegeneration. Proc Natl Acad Sci USA.

[CR63] Marini K, Mahlknecht P, Tutzer F, Stockner H, Gasperi A, Djamshidian A, Willeit P, Kiechl S, Willeit J, Rungger G, Noyce AJ, Schrag A, Poewe W, Seppi K (2020). Application of a simple Parkinson's disease risk score in a longitudinal population-based cohort. Mov Disord.

[CR64] Masellis M, Collinson S, Freeman N, ADAGIO investigators (2016). Dopamine D2 receptor gene variants and response to rasagiline in early Parkinson's disease: a pharmacogenetic study. Brain.

[CR65] McFarthing K, Rafaloff G, Baptista M, Mursaleen L, Fuest R, Wyse RK, Stott SRW (2022). Parkinson's disease drug therapies in the clinical trial pipeline: 2022 update. J Parkinsons Dis.

[CR66] Meredith GE, Rademacher DJ (2011). MPTP mouse models of Parkinson's disease: an update. J Parkinsons Dis.

[CR67] Metta V, Leta V, Mrudula KR (2022). Gastrointestinal dysfunction in Parkinson's disease: molecular pathology and implications of gut microbiome, probiotics, and fecal microbiota transplantation. J Neurol.

[CR68] Montefusco O, Assini MC, Missale C (1983). Insulin-mediated effects of glucose on dopamine metabolism. Acta Diabetol Lat.

[CR69] Moisan F, Kab S, Mohamed F, Canonico M, Le Guern M, Quintin C, Carcaillon L, Nicolau J, Duport N, Singh-Manoux A, Boussac-Zarebska M, Elbaz A (2016). Parkinson disease male-to-female ratios increase with age: French nationwide study and meta-analysis. J Neurol Neurosurg Psychiatry.

[CR70] Moreno-García A, Kun A, Calero M, Calero O (2021). The neuromelanin paradox and its dual role in oxidative stress and neurodegeneration. Antioxidants (basel).

[CR71] Mori F, Nishie M, Kakita A, Yoshimoto M, Takahashi H, Wakabayashi K (2006). Relationship among alpha-synuclein accumulation, dopamine synthesis, and neurodegeneration in Parkinson disease substantia nigra. J Neuropathol Exp Neurol.

[CR72] Nalls MA, Blauwendraat C, Vallerga CL, Heilbron K, Bandres-Ciga S, Chang D, Tan M, Kia DA, Noyce AJ, Xue A, Bras J, Young E, von Coelln R, Simón-Sánchez J, Schulte C, Sharma M, Krohn L, Pihlstrøm L, Siitonen A, Iwaki H, Leonard H, Faghri F, Gibbs JR, Hernandez DG, Scholz SW, Botia JA, Martinez M, Corvol JC, Lesage S, Jankovic J, Shulman LM, Sutherland M, Tienari P, Majamaa K, Toft M, Andreassen OA, Bangale T, Brice A, Yang J, Gan-Or Z, Gasser T, Heutink P, Shulman JM, Wood NW, Hinds DA, Hardy JA, Morris HR, Gratten J, Visscher PM, Graham RR, Singleton AB (2019) System Genomics of Parkinson's Disease Consortium; International Parkinson's Disease Genomics Consortium. Identification of novel risk loci, causal insights, and heritable risk for Parkinson's disease: a meta-analysis of genome-wide association studies. Lancet Neurol 18(12):1091–1102. 10.1016/S1474-4422(19)30320-5 10.1016/S1474-4422(19)30320-5 PMC8422160 31701892

[CR73] Naoi M, Maruyama W, Shamoto-Nagai M (2020). Rasagiline and selegiline modulate mitochondrial homeostasis, intervene apoptosis system and mitigate α-synuclein cytotoxicity in disease-modifying therapy for Parkinson’s disease. J Neural Transm (vienna).

[CR74] Nishikawa N, Murata M, Hatano T, Mukai Y, Saitoh Y, Sakamoto T, Hanakawa T, Kamei Y, Tachimori H, Hatano K, Matsuda H, Taruno Y, Sawamoto N, Kajiyama Y, Ikenaka K, Kawabata K, Nakamura T, Iwaki H, Kadotani H, Sumi Y, Inoue Y, Hayashi T, Ikeuchi T, Shimo Y, Mochizuki H, Watanabe H, Hattori N, Takahashi Y, Takahashi R; Japan Parkinson's Progression Markers Initiative (J-PPMI) study group (2022) Idiopathic rapid eye movement sleep behavior disorder in Japan: an observational study. Parkinsonism Relat Disord. 129–135. 10.1016/j.parkreldis.2022.08.011. 10.1016/j.parkreldis.2022.08.011 36113390

[CR75] Nuber S, Rajsombath M, Minakaki G, Winkler J, Müller CP, Ericsson M, Caldarone B, Dettmer U, Selkoe DJ (2018). Abrogating Native α-synuclein tetramers in mice causes a L-DOPA-responsive motor syndrome closely resembling Parkinson's disease. Neuron.

[CR76] Obeso JA, Stamelou M, Goetz CG (2017). Past, present, and future of Parkinson's disease: a special essay on the 200th Anniversary of the Shaking Palsy. Mov Disord.

[CR77] Pagano G, Polychronis S, Wilson H, Giordano B, Ferrara N, Niccolini F, Politis M (2018). Diabetes mellitus and Parkinson disease. Neurology.

[CR78] Parkkinen L, Kauppinen T, Pirttilä T, Autere JM, Alafuzoff I (2005). Alpha-synuclein pathology does not predict extrapyramidal symptoms or dementia. Ann Neurol.

[CR79] Parkkinen L, O'Sullivan SS, Collins C, Petrie A, Holton JL, Revesz T, Lees AJ (2011). Disentangling the relationship between lewy bodies and nigral neuronal loss in Parkinson's disease. J Parkinsons Dis.

[CR80] Peter I, Dubinsky M, Bressman S, Park A, Lu C, Chen N, Wang A (2018). Anti-tumor necrosis factor therapy and incidence of Parkinson disease among patients with inflammatory bowel disease. JAMA Neurol.

[CR81] Pont-Sunyer C, Tolosa E, Caspell-Garcia C, Coffey C, Alcalay RN, Chan P, Duda JE, Facheris M, Fernández-Santiago R, Marek K, Lomeña F, Marras C, Mondragon E, Saunders-Pullman R, Waro B (2017) LRRK2 Cohort Consortium. The prodromal phase of leucine-rich repeat kinase 2-associated Parkinson disease: Clinical and imaging Studies. Mov Disord 32(5):726–738. 10.1002/mds.26964 10.1002/mds.26964 28370517

[CR82] Powers KM, Smith-Weller T, Franklin GM, Longstreth WT, Swanson PD, Checkoway H (2006). Diabetes, smoking, and other medical conditions in relation to Parkinson's disease risk. Parkinsonism Relat Disord.

[CR83] Raunio A, Kaivola K, Tuimala J, Kero M, Oinas M, Polvikoski T, Paetau A, Tienari PJ, Myllykangas L (2019). Lewy-related pathology exhibits two anatomically and genetically distinct progression patterns: a population-based study of Finns aged 85. Acta Neuropathol.

[CR84] Rehman MU, Sehar N, Dar NJ, Khan A, Arafah A, Rashid S, Rashid SM, Ganaie MA (2023). Mitochondrial dysfunctions, oxidative stress and neuroinflammation as therapeutic targets for neurodegenerative diseases: an update on current advances and impediments. Neurosci Biobehav Rev.

[CR85] Riederer P, Monoranu C, Strobel S, Iordache T, Sian-Hülsmann J (2021). Iron as the concert master in the pathogenic orchestra playing in sporadic Parkinson's disease. J Neural Transm (vienna).

[CR86] Salkovic-Petrisic M, Knezovic A, Hoyer S, Riederer P (2013). What have we learned from the streptozotocin-induced animal model of sporadic Alzheimer's disease, about the therapeutic strategies in Alzheimer's research. J Neural Transm (vienna).

[CR87] Savica R, Rocca WA, Ahlskog JE (2010). When does Parkinson disease start?. Arch Neurol.

[CR88] Selikhova M, Williams DR, Kempster PA, Holton JL, Revesz T, Lees AJ (2009). A clinico-pathological study of subtypes in Parkinson's disease. Brain.

[CR89] Sian J, Dexter DT, Lees AJ, Daniel S, Agid Y, Javoy-Agid F, Jenner P, Marsden CD (1994). Alterations in glutathione levels in Parkinson's disease and other neurodegenerative disorders affecting basal ganglia. Ann Neurol.

[CR90] Sommer A, Marxreiter F, Krach F, Fadler T, Grosch J, Maroni M, Graef D, Eberhardt E, Riemenschneider MJ, Yeo GW, Kohl Z, Xiang W, Gage FH, Winkler J, Prots I, Winner B (2018). Th17 lymphocytes induce neuronal cell death in a human iPSC-based model of Parkinson's disease. Cell Stem Cell.

[CR91] Stokholm MG, Danielsen EH, Hamilton-Dutoit SJ, Borghammer P (2016). Pathological α-synuclein in gastrointestinal tissues from prodromal Parkinson disease patients. Ann Neurol.

[CR92] Sulzer D, Alcalay RN, Garretti F, Cote L, Kanter E, Agin-Liebes J, Liong C, McMurtrey C, Hildebrand WH, Mao X, Dawson VL, Dawson TM, Oseroff C, Pham J, Sidney J, Dillon MB, Carpenter C, Weiskopf D, Phillips E, Mallal S, Peters B, Frazier A, Lindestam Arlehamn CS, Sette A (2017). T cells from patients with Parkinson's disease recognize α-synuclein peptides. Nature.

[CR93] Surmeier DJ, Obeso JA, Halliday GM (2017). Parkinson's disease is not simply a prion disorder. J Neurosci.

[CR94] Svensson E, Horváth-Puhó E, Thomsen RW, Djurhuus JC, Pedersen L, Borghammer P, Sørensen HT (2015). Vagotomy and subsequent risk of Parkinson's disease. Ann Neurol.

[CR95] Takanashi M, Li Y, Hattori N (2016). Absence of Lewy pathology associated with PINK1 homozygous mutation. Neurology.

[CR96] Tanei ZI, Saito Y, Ito S, Matsubara T, Motoda A, Yamazaki M, Sakashita Y, Kawakami I, Ikemura M, Tanaka S, Sengoku R, Arai T, Murayama S (2021). Lewy pathology of the esophagus correlates with the progression of Lewy body disease: a Japanese cohort study of autopsy cases. Acta Neuropathol.

[CR97] Toffa S, Kunikowska GM, Zeng BY, Jenner P, Marsden CD (1997). Glutathione depletion in rat brain does not cause nigrostriatal pathway degeneration. J Neural Transm (vienna).

[CR98] Tribl F, Gerlach M, Marcus K, Asan E, Tatschner T, Arzberger T, Meyer HE, Bringmann G, Riederer P (2005). “Subcellular proteomics” of neuromelanin granules isolated from the human brain. Mol Cell Proteom.

[CR99] Uyar M, Lezius S, Buhmann C, Pötter-Nerger M, Schulz R, Meier S, Gerloff C, Kuhle J, Choe CU (2022). Diabetes, glycated hemoglobin (HbA1c), and neuroaxonal damage in Parkinson's disease (MARK-PD Study). Mov Disord.

[CR100] Van Den Berge N, Ferreira N, Gram H, Mikkelsen TW, Alstrup AKO, Casadei N, Tsung-Pin P, Riess O, Nyengaard JR, Tamgüney G, Jensen PH, Borghammer P (2019). Evidence for bidirectional and trans-synaptic parasympathetic and sympathetic propagation of alpha-synuclein in rats. Acta Neuropathol.

[CR101] Villumsen M, Aznar S, Pakkenberg B, Jess T, Brudek T (2019). Inflammatory bowel disease increases the risk of Parkinson's disease: a Danish nationwide cohort study 1977–2014. Gut.

[CR102] von Coelln R, Gruber-Baldini AL, Reich SG, Armstrong MJ, Savitt JM, Shulman LM (2021). The inconsistency and instability of Parkinson's disease motor subtypes. Parkinsonism Relat Disord.

[CR103] Walker Z, Costa DC, Walker RW, Lee L, Livingston G, Jaros E, Perry R, McKeith I, Katona CL (2004). Striatal dopamine transporter in dementia with Lewy bodies and Parkinson disease: a comparison. Neurology.

[CR104] Weintraub D, Aarsland D, Chaudhuri KR, Dobkin RD, Leentjens AF, Rodriguez-Violante M, Schrag A (2022). The neuropsychiatry of Parkinson's disease: advances and challenges. Lancet Neurol.

[CR105] Whone A (2022). Monoclonal antibody therapy in Parkinson's disease—the end?. N Engl J Med.

[CR106] Wüllner U, Seyfried J, Groscurth P, Beinroth S, Winter S, Gleichmann M, Heneka M, Löschmann P, Schulz JB, Weller M, Klockgether T (1999). Glutathione depletion and neuronal cell death: the role of reactive oxygen intermediates and mitochondrial function. Brain Res.

[CR107] Wüllner U, Kaut O, deBoni L, Piston D, Schmitt I (2016). DNA methylation in Parkinson's disease. J Neurochem.

[CR108] Yacoubian TA, Fang YD, Gerstenecker A, Amara A, Stover N, Ruffrage L, Collette C, Kennedy R, Zhang Y, Hong H, Qin H, McConathy J, Benveniste EN, Standaert DG (2023). Brain and systemic inflammation in de novo PARKINSON'S Disease. Mov Disord.

[CR109] Zhang L, Zhang L, Li L, Hölscher C (2019). Semaglutide is neuroprotective and reduces α-synuclein levels in the chronic MPTP mouse model of Parkinson's disease. J Parkinsons Dis.

[CR110] Zittel S, Uyar M, Lezius S, Gerloff C, Choe CU (2021). HbA1c and motor outcome in Parkinson's disease in the mark-PD study. Mov Disord.

